# A study on the precision of voxel- and surface-based mandibular superimposition

**DOI:** 10.12669/pjms.40.8.8441

**Published:** 2024-09

**Authors:** Gengbing Lin, Xue-ting Chen, Xie Shi

**Affiliations:** 1 Gengbing Lin, Department of Stomatology, Fujian Provincial People’s Hospital, Affiliated People’s Hospital of Fujian University of Traditional Chinese Medicine, Fuzhou 350004, Fujian, China; 2 Xue-ting Chen, Department of Orthodontics, Fujian Key Laboratory of Oral Diseases & Fujian Provincial Engineering Research Center of Oral Biomaterial & Stomatological Key lab of Fujian College and University, School and Hospital of Stomatology, Fujian Medical University & Institute of Stomatology, Fujian Medical University, Fuzhou 350004, Fujian, China; 3 Xie Shi, Department of Orthodontics, Fujian Key Laboratory of Oral Diseases & Fujian Provincial Engineering Research Center of Oral Biomaterial & Stomatological Key lab of Fujian College and University, School and Hospital of Stomatology, Fujian Medical University & Institute of Stomatology, Fujian Medical University, Fuzhou 350004, Fujian, China

**Keywords:** Mandibular superimposition, Voxel‐based superimposition, Surface‐based superimposition

## Abstract

**Objective:**

To apply the more accurate technique for mandibular superimposition and provide a valuable reference for the assessment of mandibular tooth movement and condylar remodeling before and after orthodontic treatment.

**Methods:**

This retrospective study involved 38 adult patients who underwent two cone beam computer tomography (CBCT) scans at different stages of treatment at Fujian Provincial People’s Hospital between September, 2020 and December, 2022. The software Dolphin was used for mandible segmentation, enabling voxel‐based mandibular superimposition with the mandibular ramus as the reference region. The Geomagic Wrap software was employed to process surface‐based mandibular superimposition with the mandibular ramus as a reference. Additionally, the voxel and surface‐based methods were compared for precision, with the mandibular ramus being the reference.

**Results:**

After voxel‐based mandibular superimposition using the mandibular ramus as a reference, with all measurement errors (< 0.20 mm). In contrast, the results of surface‐based mandibular superimposition with the same reference, and the measurement errors were all less than 0.10 mm. The Wilcoxon signed‐rank test revealed statistically significant differences between AS1 and BS1, AS2 and BS2, AS3 and BS3, and AS4 and BS4 (all r< 0.05). Moreover, the absolute mean distances of AS1-AS4 were all greater than those of BS1-BS4.

**Conclusion:**

All mandibular superimposition procedures, including the voxel‐ and surface‐based ones using the mandibular ramus as a reference, have acceptable surface errors (< 0.20 mm), indicating the good reliability of these techniques. Under the specified conditions, surface‐based mandibular superimposition appears to yield a higher degree of precision compared with the voxel‐based technique.

## INTRODUCTION

Lateral cephalograms were widely used in the evaluation of orthodontic treatment outcomes and craniofacial growth development and have been replaced by the now preferred alternative cone‐beam computed tomography (CBCT) due to its limitations that affect the degree of precision as a two-dimensional(2D) examination, including overlapping of craniofacial structures, irregular magnification, and landmark identification errors.^1^ Cone beam computer tomography(CBCT) has rapidly advanced in oral medicine and is now primarily used for three-dimensional(3D) diagnosis in orthodontic clinics in addition to such applications as locating the position of an impacted tooth, measuring periodontal bone mass, evaluating arch expansion treatment outcomes, determining tooth movement, and assessing temporomandibular joint reconstruction and airways.^2^,^3^ By comparing pre- and post-treatment 3D images, CBCT also allows for the reconstruction of dental, skeletal, and craniofacial morphologies and visualization of craniofacial changes in the evaluation of growth development and treatment outcomes.^4^

Anatomical landmark, surface-based, and voxel-based superimposition are the most frequently used 3D techniques. Landmark superimposition involves selecting specific anatomical landmarks and forming reference lines or planes to compare two sets of 3D scans, which is similar to the traditional method using lateral cephalograms.^5^ In contrast, surface-based superimposition relies on high-quality 3D curved surfaces^6^, while the voxel-based technique works by matching 3D images taken at different time points via making adjustments to all voxels of an image according to the other to ensure the best fit for each voxel.^7^ All these methods-landmark, surface, and voxel overlapping-have proven reliable in previous research. However, landmark superimposition shows a lower degree of precision due to human errors in landmark identification and spatial errors in 3D localization. Almukhtar et al.^8^ reported both voxel- and surface-based superimposition to be reliable, and the surface-based superimposition of hard tissues being as accurate as the voxel-based method.

Currently, there is no gold standard for CBCT superimposition, and only very limited studies have compared the precision of different superimposition methods. Therefore, the present study investigated the reliability and compared the precision of voxel- and surface-based superimposition using the mandibular ramus as a reference, aiming to provide a solid theoretical foundation for the clinical 3D visual evaluation of tooth movement and condylar remodeling before and after treatment.

## METHODS

This was a retrospective study. A total of 38 patients who underwent orthodontic treatment at Fujian Provincial People’s Hospital between September, 2020 and December, 2022 received two or more CBCT scans during the treatment course, with a minimum interval of one year between the scans.

### Ethical Approval:

The study was approved by the Institutional Ethics Committee of Fujian Medical University & Institute of Stomatology, Fujian Medical University on December 9, 2021 (No. [2020]87), and written informed consent was obtained from all participants.

The CBCT images were captured by an experienced in-house radiologist using the i-CAT CBCT system (i-CAT, LLC, USA). The scanning parameters were set as follows: voltage= 120 kV, current = 5 mA, exposure time = seven seconds, slice thickness = 0.20 mm, and field of view= 13 cm. Prior to the scan, the patient’s head position was adjusted to align the orbital-ear plane parallel to the ground, with the patient looking straight ahead. The patients were instructed to bite naturally and maintain a steady intercuspal position without swallowing or talking during the scan. Clear CBCT images were collected, exported, and saved in the Digital Imaging and Communications in Medicine (DICOM) file format. In total, 76 CBCT scans were obtained from the 38 participants, comprising 38 image datasets. The CBCT scan taken at an earlier time point was referred to as CT1, while the scan taken at a later time point was referred to as CT2.

### CBCT data for mandibular reconstruction:

**Fig.1:**
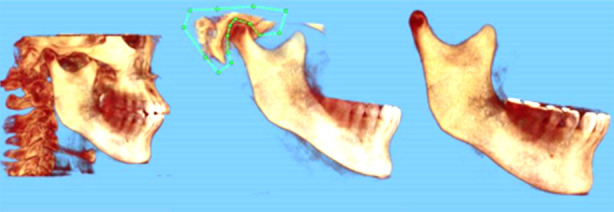
Segmentation of right mandible via volume reconstruction in Dolphin.

The CBCT data from CT1 and CT2 were imported into Dolphin for segmentation of the mandibular structure via volume reconstruction (Fig.1). This involved the complete extraction of the 3D data of the mandibular condyle from CBCT scans, and only the right mandible was used for superimposition. The DICOM data of the right mandible from CT1 was labeled as T1, while the DICOM data from CT2 was labeled as T2. These files were exported and saved in the DICOM file format.

### Mandibular superimposition:

**Fig.2:**
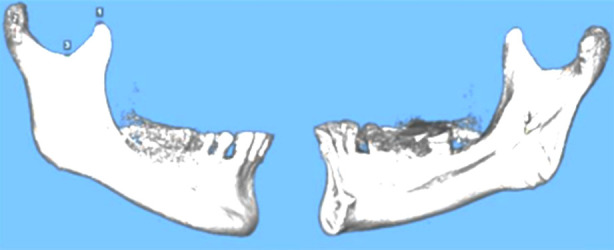
Four landmark points for rough alignment of mandible models.

**Fig.3:**
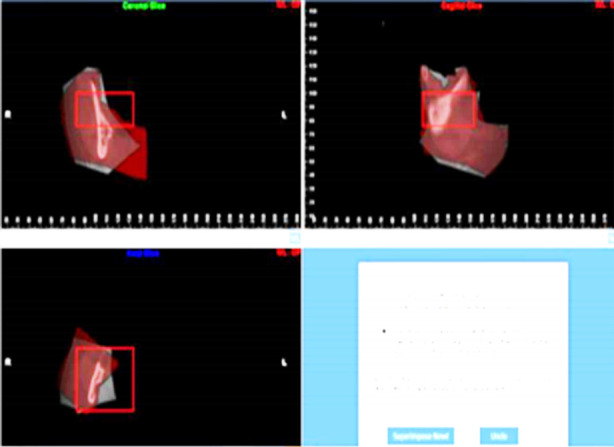
Voxel-based superimposition by reference to the right mandibular ramus.

T1 and T2 were separately imported into Dolphin, with consistent grayscale settings to ensure optimal visualization of the condylar structure. The software supports voxel-based superimposition (Method A), and the process includes two steps:

- initial superimposition (Fig.2): four landmark points were chosen to roughly align the models, namely the outermost point of the condyle, the lowest point of the sigmoid notch, the highest point of the coracoid process, and the entrance of the mandibular canal;
- voxel-based superimposition (Fig.3): The mandibular ramus, specifically the region below the inferior sigmoid notch to the entrance of the mandibular canal, was chosen as the reference for voxel-based superimposition. The initial superimposition can facilitate alignment and reduce the time required for voxel-based superimposition. After superimposition, T1 and T2 were maintained in the same relative position. The models were exported and saved in the stereolithography (STL) file format, preserving the same grayscale values.

**Fig.4:**
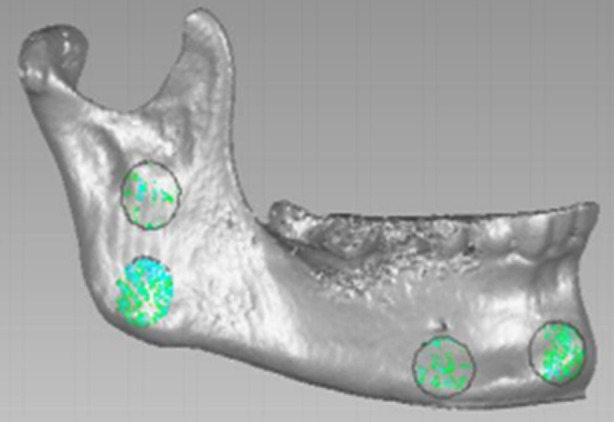
Measurement of post-superimposition absolute average distances.

The overlapped models of T1 and T2 in the STL format were imported into Geomagic Wrap to visually assess the quality of T1-T2 superimposition using a deviation map (1.5b), with a uniform green color indicating a satisfactory overlap. If necessary, the superimposition process was repeated to ensure accurate alignment. The absolute average distance between the surfaces of the T1 and T2 3D models was measured using the iterative closest point algorithm. Four circular regions - each covering an area of approximately 1 cm² - at the mid-symphysis, the inferior mental foramen, the mandibular angle, and the center of the mandibular ramus. These regions were denoted as AS1, AS2, AS3, and AS4, respectively. The surface absolute average distance between each of these four regions in the two aligned models was measured via surface deviation analysis in Geomagic Wrap (Fig.4).

### Surface of 3D model for mandibular reconstruction:

After reconstruction using the DICOM-format T1 and T2 of the 38 samples, 3D surfaces models of T1 and T2 were reconstructed via surface reconstruction in Dolphin, with both models using the same grayscale threshold, and the models were exported and saved in STL format.

### Surface-based mandibular superimposition:

**Fig.5:**
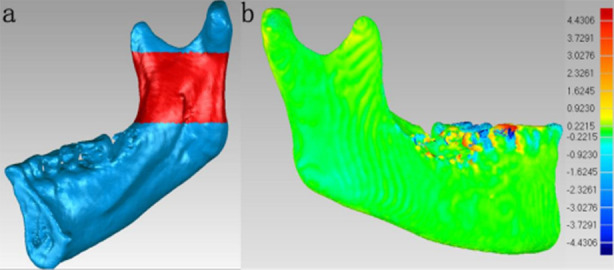
Reference region and evaluation of mandibular superimposition.

The STL-format surface models of T1 and T2 were imported into Geomagic Wrap to proceed with surface-based superimposition (Method B) via “n-point alignment” plus “best-fit alignment” using the right mandible models generated at different time points of the treatment course. Details of the superimposition are described as follows:

*N-point alignment:* Four landmark points were chosen to align the models, including the outermost condyle, the lowest point of the sigmoid notch, the highest point of the coronoid process, and the entrance of the mandibular canal. The region below the sigmoid notch to the entrance of the mandibular canal was chosen as the reference for best-fit alignment and superimposition (Fig.5a). N-point alignment was conducted to ensure efficient and accurate alignment between the two models. A deviation map (Fig.5b) was plotted to visually evaluate the quality of superimposition between T1 and T2.

Four circular regions of approximately 1cm² each were denoted as BS1, BS2, BS3, and BS4 on the superimposed mandible models, including the mid-symphysis, the region below the mandibular foramen, the mandibular angle, and the center of the mandibular ramus. The surface absolute average distance within each of these four regions was determined through the surface deviation analysis.

A sample size analysis was conducted to determine the number of cases needed for the study. With the settings of a significance level (α) of 0.05 (two-tailed test) and a power (1-β) of 0.8, the preliminary experiment yielded a mean surface error of 0.255 and a standard deviation of 0.520 in the mid-symphysis (S1) region after voxel-based superimposition. On this basis, the sample size estimation in the software PASS indicated that the minimum sample size should be 35 cases per group. In the current study, 38 subjects were included, which met the sample size requirement.

To assess the consistency of the measurements, all procedures were performed by one researcher, and all measurements were taken in triplicate at a two-week interval. The intraclass correlation coefficient (ICC) was adopted to assess the consistency of the triplicate measurements for both voxel- and surface-based superimposition in the mid-symphysis (S1) region, with an ICC value greater than 0.75 indicative of good consistency in the establishment of 3D models and measurement accuracy.

### Statistical analysis:

The software SPSS 22.0 was used for statistical analysis. The significance level was set at α = 0.05, and a p-value less than 0.05 was considered statistically significant.For each superimposition method, the means of the four measurement regions were separately analyzed, and a mean value smaller than the CBCT slice thickness (0.20 mm) indicated that the superimposition was reliable. The Shapiro-Wilk test was undertaken to assess data normality. Normally distributed data were presented as “mean ± standard deviation,” while non-normally distributed data were expressed as “median (P25, P75).” The paired-sample t-test was used for comparison if both surface absolute average distances from the two superimposition methods followed a normal distribution. On the other hand, the Wilcoxon signed-rank test was used for the comparison of non-normally distributed data. The Wilcoxon signed-rank test was employed to compare the measured values between voxel- and surface-based superimposition methods based on the specified regions.

## RESULTS

Reliability analysis of voxel- and surface-based superimposition with mandibular ramus as a reference. After voxel-based superimposition, the measured absolute average distance was 0.103 (0.055, 0.181) mm for AS1, 0.115 ± 0.074 mm for AS2, 0.048 (0.028, 0.121) mm for AS3, and 0.051 (0.037, 0.091) mm for AS4. Table-I. After surface-based superimposition, the measured absolute average distance was 0.064 (0.046, 0.087) mm for BS1, 0.057 (0.025, 0.095) mm for BS2, 0.049 ± 0.032 mm for BS3, and 0.037 (0.012, 0.056) mm for BS4. Table-II. All measured values in both methods for all regions were less than 0.20 mm, indicating a high degree of reliability.

**Table-I:** Measured values of absolute average distances after voxel-based superimposition.

Item	Mean	SD	Min.	Max.	P25	P50	P75
AS1 (mm)	0.135	0.103	0.001	0.360	0.055	0.103	0.181
AS2 (mm)	0.115	0.074	0.011	0.300	0.049	0.105	0.174
AS3 (mm)	0.081	0.076	0.000	0.370	0.028	0.048	0.121
AS4 (mm)	0.073	0.059	0.000	0.250	0.037	0.051	0.091

**Table-II:** Measured values of absolute average distances after surface-based superimposition.

Item	Mean	SD	Min.	Max.	P25	P50	P75
BS1 (mm)	0.077	0.059	0.001	0.310	0.046	0.064	0.087
BS2 (mm)	0.072	0.058	0.000	0.270	0.025	0.057	0.095
BS3 (mm)	0.049	0.032	0.000	0.130	0.023	0.038	0.068
BS4 (mm)	0.040	0.032	0.000	0.140	0.012	0.037	0.056

**Fig.6:**
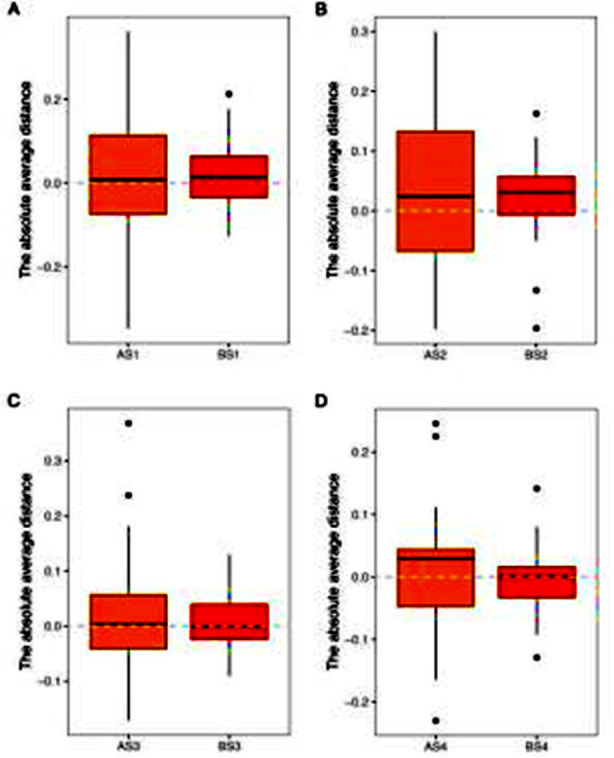
Comparison of surface absolute average distances.

The Wilcoxon signed-rank test results revealed statistically significant differences between the voxel- and surface-based methods for AS1 and BS1, AS2 and BS2, AS3 and BS3, and AS4 and BS4 (all *P*< 0.05). Additionally, the surface average distances of AS1, AS2, AS3, and AS4 were found to be greater than those of BS1, BS2, BS3, and BS4, respectively. Table-III and Fig.6.

**Table-III:** Comparison of surface absolute average distances.

Item	Voxel-based superimposition (Method A)	Surface-based superimposition (Method B)	Z-value	P-value
S1 (mm)	0.103(0.055,0.181)	0.064(0.046,0.087)	-2.226	0.026*
S2 (mm)	0. 115±0.074	0.057(0.025,0.095)	-2.560	0.010*
S3 (mm)	0.048(0.028,0.121)	0.049±0.032	-2.067	0.039*
S4 (mm)	0.051(0.037,0.091)	0.037(0.012,0.056)	-3.017	0.003**

## DISCUSSION

The results of statistical analysis showed that the surface average absolute distances for both voxel- and surface-based superimposition were less than 0.20 mm. Notably, the measured values were all less than 0.10 mm except the average absolute distances for the mid-symphysis (0.103 mm) and the region below the mandibular foramen (0.115 mm) in voxel-based superimposition. These findings suggest that both methods are precise and reliable, and surface-based superimposition demonstrates a modestly higher degree of precision compared with the voxel-based method, with the differences between these methods showing statistical significance. This can be explained by the reduced voxel correspondence strength due to the segmentation and reconstruction of the mandible, as well as the transformation of storage format after superimposition. Besides, the hard tissue surface contains highly detailed information that enhances the performance of surface-based superimposition. These opinions are consistent with the findings of several previous studies.^9^-^11^ However, it is worth noting that the comparative study by Almukhtar et al.^8^ demonstrates no significant difference in the degree of precision between voxel-and surface-based superimposition using the anterior cranial base as the reference region. For the surface-based method, it is highly unstable to measure the distance between the overlapping surfaces of the two models.

In their study, the software VRMesh (Virtual Grid, Bellevue City, WA, USA) was used for surface-based superimposition, while Maxilim (Medicin-Medical Image Computing, Mechelen, Belgium) was employed to perform voxel-based superimposition. This may also explain the differences in the superimposition results.

In orthodontic clinical practice, the superimposition of pre- and post-treatment imaging data is required to evaluate the growth direction of craniofacial structures or the treatment outcomes. Traditionally, 2D radiographs were extensively used for this purpose.^12^ However, relying solely on 2D measurements can result in information loss since 2D superimposition doesn’t account for 3D changes and lateral dimensions.^13^

To address this limitation, 3D superimposition has been widely studied, and various anatomical structures have been proposed for analysis. For instance, 3D maxillary digital models (MDMs) based on the palatal vault have been used for the evaluation of tooth movement in orthodontic practice. Garib et al.^14^ compared 3D MDM-based superimposition with CBCT-based maxillary superimposition and concluded that the MDM method is effective and reliable for evaluating tooth movement in adults, although slightly less reliable than maxillary superimposition using CBCT. MDM-based superimposition does not apply to the mandibular dentition because mandible models have no stable structures. In CBCT-based superimposition, the cranial base has been suggested as a reference for evaluating overall facial growth and treatment outcomes^15^ considering that the cranial base has completed 90% of its growth by 4-5 years.^16^ After extensive research on different regions of the cranial base for superimposition, the anterior cranial base has been identified as the optimal fitting region.^15^,^17^ However, considering that the temporomandibular joint can cause significant changes in the mandibular position during orthodontic treatment, it is challenging to observe any changes in the mandibular dentition or achieve condylar remodeling via superimposition of the anterior cranial base. This underpins the need for further research on 3D mandibular overlap methods. In this study, only the right mandible was chosen for superimposition because using the bilateral mandibles might affect sample independence due to mandibular symmetry.^18^

Voxel-based superimposition processes raw data via the grayscale intensities of voxels in DICOM images, offsetting the grayscale values of the surrounding anatomical structures with the embedded anatomical structures, while surface-based superimposition generates 3D surface mesh models by rendering DICOM images and superimpose two models with the data obtained from the grid-based terrain of the 3D models. Both methods allow for superimposition based on the algorithm called iterative closest point.^19^ For surface-based superimposition, the distance between two surfaces is minimized through the optimal translation and rotation of 3D shapes. As to voxel-based superimposition, the optimal translation and rotation between two image volumes can be determined using a specified percentage of voxels and the mean square difference by subtracting the grayscale intensities of the voxels from those of the superimposed image volume.

### Limitations of this study:

It includes a sample size of this study was small, with limited clinical data available and limited persuasive conclusions. Further intervention trials are needed in the future to confirm these results.

## CONCLUSIONS

The voxel-and surface-based mandibular superimposition techniques using the mandibular ramus as the reference region both demonstrate good reliability, with the surface errors of all measured regions less than 0.10 mm except the mid-symphysis (0.103 mm) and the region below the mandibular foramen (0.115 mm) in voxel-based superimposition. Compared with surface-based superimposition, the voxel-based technique has a higher degree of precision. as indicated by the surface errors in the measurement regions.

### Authors’ Contributions:

**GL** carried out the studies, participated in collecting data, drafted the manuscript, are responsible and accountable for the accuracy or integrity of the work.

**XC** performed the statistical analysis and participated in its design.

**XS** participated in acquisition, analysis, or interpretation of data and draft the manuscript.

All authors read and approved the final manuscript.
